# Qualitative and Quantitative MRI Analysis in IDH1 Genotype Prediction of Lower-Grade Gliomas: A Machine Learning Approach

**DOI:** 10.1155/2021/1235314

**Published:** 2021-01-22

**Authors:** Mengqiu Cao, Shiteng Suo, Xiao Zhang, Xiaoqing Wang, Jianrong Xu, Wei Yang, Yan Zhou

**Affiliations:** ^1^Department of Radiology, Renji Hospital, School of Medicine, Shanghai Jiao Tong University, Shanghai 200127, China; ^2^Biomedical Instrument Institute, School of Biomedical Engineering, Shanghai Jiao Tong University, Shanghai 200030, China; ^3^Zhuhai Precision Medical Center, Zhuhai People's Hospital (Zhuhai Hospital Affiliated with Jinan University), Zhuhai 519000, China; ^4^Guangdong Provincial Key Laboratory of Medical Image Processing, School of Biomedical Engineering, Southern Medical University, Guangzhou 510515, China

## Abstract

**Purpose:**

Preoperative prediction of isocitrate dehydrogenase 1 (IDH1) mutation in lower-grade gliomas (LGGs) is crucial for clinical decision-making. This study aimed to examine the predictive value of a machine learning approach using qualitative and quantitative MRI features to identify the IDH1 mutation in LGGs.

**Materials and Methods:**

A total of 102 LGG patients were allocated to training (*n* = 67) and validation (*n* = 35) cohorts and were subject to Visually Accessible Rembrandt Images (VASARI) feature extraction (23 features) from conventional multimodal MRI and radiomics feature extraction (56 features) from apparent diffusion coefficient maps. Feature selection was conducted using the maximum Relevance Minimum Redundancy method and 0.632+ bootstrap method. A machine learning model to predict IDH1 mutation was then established using a random forest classifier. The predictive performance was evaluated using receiver operating characteristic (ROC) curves.

**Results:**

After feature selection, the top 5 VASARI features were enhancement quality, deep white matter invasion, tumor location, proportion of necrosis, and T1/FLAIR ratio, and the top 10 radiomics features included 3 histogram features, 3 gray-level run-length matrix features, and 3 gray-level size zone matrix features and one shape feature. Using the optimal VASARI or radiomics feature sets for IDH1 prediction, the trained model achieved an area under the ROC curve (AUC) of 0.779 ± 0.001 or 0.849 ± 0.008 on the validation cohort, respectively. The fusion model that integrated outputs of both optimal VASARI and radiomics models improved the AUC to 0.879.

**Conclusion:**

The proposed machine learning approach using VASARI and radiomics features can predict IDH1 mutation in LGGs.

## 1. Introduction

Diffuse lower-grade gliomas (LGGs; World Health Organization (WHO) grade II or III) are infiltrative neoplasms which account for about 33%-45% of all adult gliomas [1, 2]. Although LGGs are usually less aggressive with better treatment response and prolonged prognosis compared with glioblastomas (WHO grade IV), many cases eventually progress to glioblastoma. Previous studies have shown that the high tumor heterogeneity in clinical behavior depends on genetics more than histology [1–3]. Therefore, the 2016 WHO classification of Tumors of the Central Nervous System integrates molecular biomarkers with histology for glioma diagnosis [4].

Isocitrate dehydrogenase (IDH) is one of the most important molecular biomarkers in gliomagenesis. In the 2016 WHO classification scheme, IDH mutation status serves as the first molecular determinant beyond histology, and accordingly, LGG is classified into IDH-mutant and IDH-wildtype entities [4]. Patients with an IDH-mutated glioma have a longer survival duration than those with an IDH-wildtype tumor. Recent evidence has also suggested that IDH may be a potential therapeutic target in IDH-mutant gliomas [5]. Therefore, preoperative prediction of IDH mutation status is crucial for prognosis and therapeutic decision-making.

MRI can facilitate glioma diagnosis in a noninvasive manner [6, 7]. Qualitative MRI analysis still remains the basis in imaging diagnosis. For interpretation accuracy and consistency, Visually Accessible Rembrandt Images (VASARI) lexicon based on conventional MRI has been proposed to describe the features and guidelines. Previous studies have shown the biological or clinical relevance of the VASARI features in gliomas. For example, Zhou et al. [6] reported that VASARI features including proportion of necrosis and lesion size were associated with IDH1 mutation status.

Quantitative MRI has emerged as a promising tool in the evaluation of gliomas as it can provide information on tumor functionality. Apparent diffusion coefficient (ADC) calculated from diffusion-weighted imaging (DWI) is one of the most clinically useful quantitative measurements [8–10]. Radiomics, a recently developed high-throughput approach, can add value to the routine MRI to a greater extent by extracting and mining a large number of imaging traits [11]. Growing evidence has revealed the feasibility and clinical implications of radiomics in the characterization of glioma phenotypes [6, 12].

We hypothesized that the use of both qualitative and quantitative MRI features could facilitate better IDH genotype discrimination. In this study, we aimed to develop a machine learning approach based on VASARI and ADC radiomics features to characterize the IDH1 mutation status in LGGs.

## 2. Materials and Methods

### 2.1. Subjects

This retrospective study was approved by the local institutional review board with a waiver of the written informed consent from patients. Patients were identified by searching the database of our institution for radiologic and histopathologic records from January 2015 to December 2018. The inclusion criteria for the study patients were as follows: (a) histologically proven LGG; (b) available IDH1 mutation records; (c) complete preoperative MRI data including native T1- and T2-weighted imaging (T1W and T2W); T2 fluid attenuation inversion recovery (FLAIR), DWI, and postcontrast T1W; and (d) sufficient image quality. Patients who had received treatment for glioma prior to MRI were excluded. Finally, 102 LGG patients (60 men and 42 women; age range, 18-77 years; mean age, 45.3 ± 16.3 years) were included for the subsequent analyses. Subjects were randomly divided into two subsets, a training cohort (*n* = 67) and a validation cohort (*n* = 35).

### 2.2. MRI

Images were acquired using a 3 Tesla MRI system (Signa HDxt; GE Medical Systems, Milwaukee, Wis, USA) with an eight-channel head coil. The protocol included native T1W, T2W, FLAIR, and DWI in the axial plane and postcontrast T1W in three orthogonal planes. Postcontrast imaging was achieved with intravenous administration of 0.1 mmol/kg dose of gadopentetate dimeglumine (Magnevist; Bayer Healthcare, Berlin, Germany). In all native sequences, the same asymmetric field of view (260 × 260 mm^2^), section thickness (5 mm), and intersection gap (20%) were used. DWI was performed before the injection of contrast material with repetition time = 4850 ms, echo time = 74 ms, acquisition matrix = 160 × 160, *b* value = 0 and 1000 sec/mm^2^, and number of averages = 2.

### 2.3. Feature Extraction

For qualitative image analysis, readings were performed on all sequences with a Digital Imaging and Communications in Medicine viewer (RadiAnt DICOM Viewer; Poznan, Poland) by two neuroradiologists (Mengqiu Cao and Yan Zhou, with 6 and 19 years of experience in neurological MRI interpretation, respectively) in consensus. Each tumor was scored according to the VASARI lexicon, which consists of 23 imaging traits related to the morphology of brain tumors. Detailed descriptions of the VASARI feature set are available in Supplementary Table S1.

For quantitative ADC analysis, segmentation of the tumor area was first manually performed using the 3D Slicer software (version 4.7; https://www.slicer.org). The tumor area was defined as the abnormal hyperintensity area on FLAIR images. The volume of interest (VOI) was generated by including all consecutive image sections containing tumor areas. Independent analysis of the segmentation labels (from 30 randomly selected subjects in the training set) by two neuroradiologists was conducted to evaluate the interobserver reliability of the segmentation. The Dice similarity coefficient (DSC) [13] was measured over the two labels per case from the two neuroradiologists. A DSC value of 0 indicates no overlap and a value of 1 corresponds to exact overlap. After registering ADC maps to FLAIR images, VOI was propagated to ADC maps. A total of 56 radiomics features were then extracted from the volumetric ADC data including 3 shape features, 13 first-order histogram features, 9 gray-level co-occurrence matrix (GLCM) features, 13 gray-level run-length matrix (GLRLM) features, 13 gray-level size zone matrix (GLSZM) features, and 5 neighborhood gray-tone difference matrix (NGTDM) features [14]. Before the feature selection process, all the radiomics features were normalized to the range of [0, 1] for standardization, so that features of different orders of magnitude could be reasonably compared. Feature extraction was performed using the Matlab software (version 2016a; MathWorks, Natick, Mass, USA). Detailed calculations of the radiomics features are provided in Supplementary Table S2.

### 2.4. Feature Selection

Our study adopted a two-step feature selection scheme to identify the most predictive variables. First, the maximum Relevance Minimum Redundancy (mRMR) method was used to select features that had the maximal mutual information with respect to the target class (maximum relevance) and minimal mutual information with respect to each other (minimum redundancy). Second, the 0.632+ bootstrap method and the area under the receiver operating characteristic curve (AUC) metric were used to explore the features with optimal discrimination performance on the training data set [14]. A random forest classifier was chosen as a statistical model in this process. According to the AUC metric, the top 5 VASARI and 10 radiomics features were finally selected for further predictive model building.

### 2.5. Machine Learning-Based Prediction

Predictive models of different orders (1–5 for VASARI features and 1–10 for radiomics features) were constructed separately on the optimal combinations of VASARI and radiomics features. Random forest classifiers were trained on the training cohort. The prediction performance was evaluated with the 0.632+ bootstrap AUC method. Sensitivity, specificity, accuracy, and AUC were calculated for each condition.

The random forest prediction models were then validated on the validation cohort. Further, the fusion model from the optimal VASARI model and radiomics model was obtained by integrating the predicted probability of both models. The weight value of fusion of the two models was set according to the weighted average fusion strategy, that was, 0.5. When analyzing a new case, we separately calculated the prediction probability of VASARI and radiomics models and, then, averaged the two values as the final prediction probability. To demonstrate the complementary roles of VASARI and radiomics features in the fusion model, the correlation analysis was performed using the Pearson correlation coefficient. The prediction performance of the fusion machine learning model was evaluated. The influence of common clinical variables including age and gender on the prediction performance was also tested.

**Figure 1:**
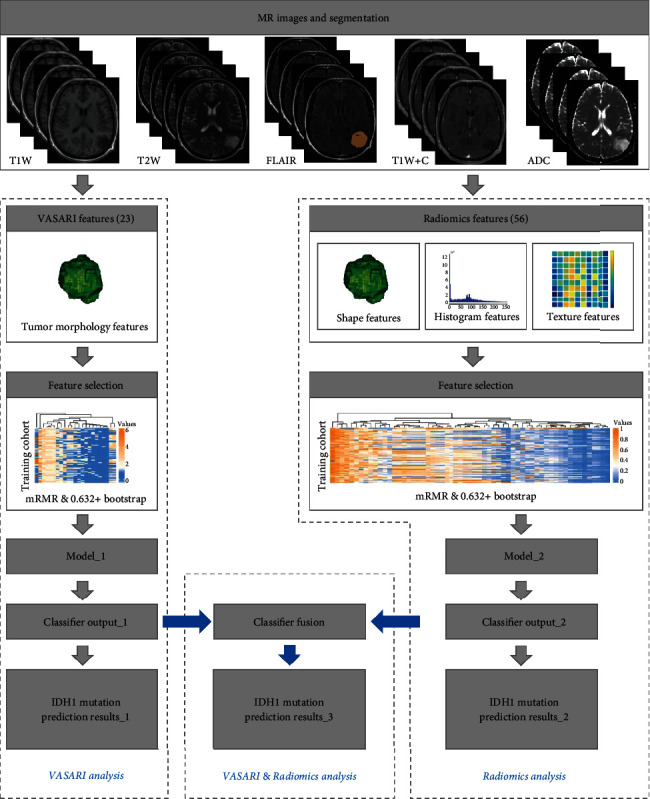
The flowchart of the experimental design of the machine learning approach.

The flowchart of the experimental design of the machine learning approach is illustrated in Figure 1. All the machine learning algorithms were implemented using the Matlab software.

### 2.6. Statistical Analysis

Comparison of categorical characteristics between groups was performed with the chi-square test or Fisher's exact test and comparison of continuous characteristics with Student's *t*-test. Receiver operating characteristic (ROC) curves were generated on the basis of the classification results of random forest models. Results with *P* values less than 0.05 were considered to indicate a significant difference. All the statistical analyses were performed using the Matlab software and IBM SPSS Statistics software (version 21; SPSS, Chicago, Ill, USA).

## 3. Results

### 3.1. Patient Characteristics

Of all the 102 LGG patients, 61 (59.8%) were diagnosed as WHO grade II glioma and 41 (40.2%) with WHO grade III. Among them, 50 (49%) and 52 (51%) patients were confirmed with IDH1-mutant and IDH1-wildtype LGG, respectively. Patient characteristics of the whole cohort, the training cohort, and the validation cohort were summarized in Table 1. No significant difference in age, gender, WHO grade, or IDH1 mutation status was noted between the training and validation cohorts (*P* > 0.05).

### 3.2. Interobserver Reliability of Segmentation

**Figure 2:**
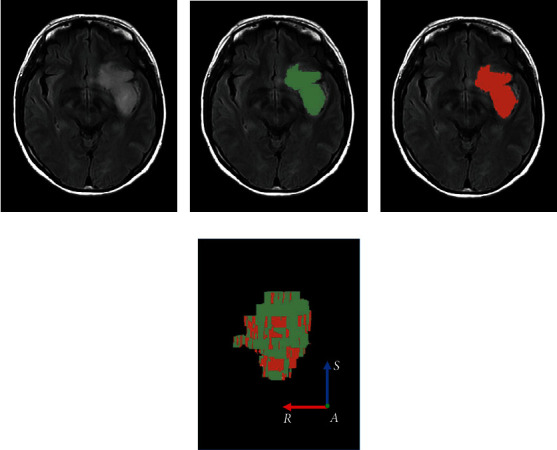
Interobserver reliability of contours between the two neuroradiologists. (a) One original section of the volumetric data. (b) Contour delineated by the first neuroradiologist. (c) Contour delineated by the second neuroradiologist. (d) Overlaid 3D volume rendering image (AP view).

Interobserver reliability analysis of the manual segmentation showed good agreement between the neuroradiologists, with a DSC score of 0.879 ± 0.046. A representative case showing the interobserver reliability of segmentation is illustrated in Figure 2.

### 3.3. IDH1 Mutation Prediction with VASARI Features

**Figure 3:**
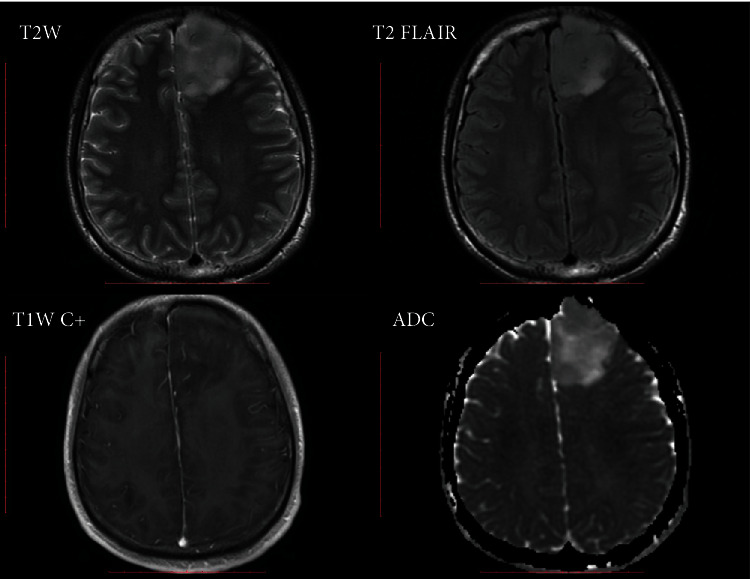
A 26-year-old man with an IDH1-mutant glioma (diffuse astrocytoma, WHO grade II). The tumor is located in the frontal lobe with no contrast enhancement, no deep white matter invasion, no necrosis, and an expansive tumor behavior (T1~FLAIR).

**Figure 4:**
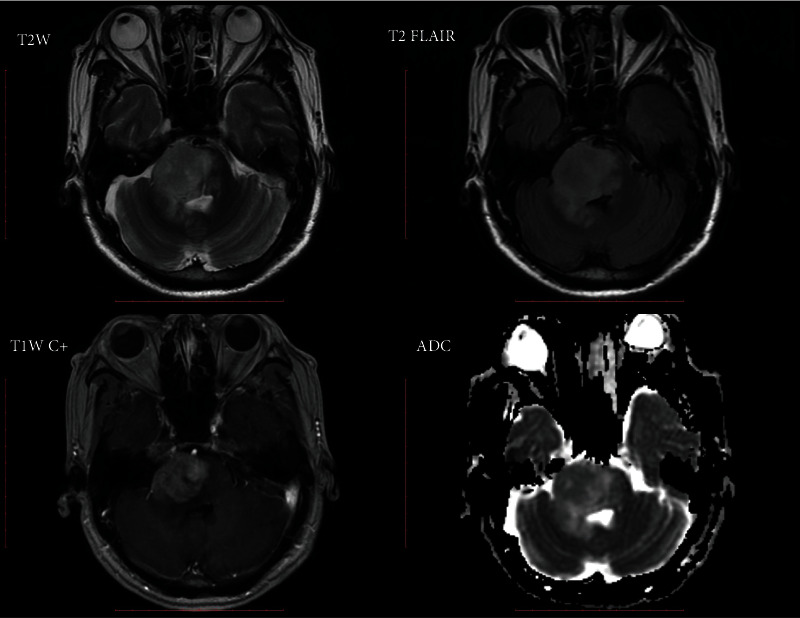
A 65-year-old man with an IDH1-wildtype glioma (diffuse astrocytoma, WHO grade II). The tumor is located in the brainstem with marked contrast enhancement, deep white matter invasion, a necrosis proportion of <33%, and a mixed tumor behavior (T1<FLAIR).

After feature selection, the top 5 VASARI features were enhancement quality, deep white matter invasion, tumor location, proportion of necrosis, and T1/FLAIR ratio (Table 2). Prediction models with orders 1 to 5 were generated by incorporating the above optimal features. On the training cohort, the highest AUC of 0.827 ± 0.031 was reached, with a sensitivity of 0.671 ± 0.058 and a specificity of 0.712 ± 0.049, respectively. Using the optimal feature set (the single enhancement quality feature), the trained model achieved an AUC of 0.779 ± 0.001 on the validation cohort, with a sensitivity of 0.718 ± 0.070, a specificity of 0.733 ± 0.100, and an accuracy of 0.726 ± 0.017, respectively. Representative cases of IDH1-mutant and IDH1-wildtype LGGs are shown in Figures 3 and 4.

### 3.4. IDH1 Mutation Prediction with Radiomics Features

In ADC radiomics analysis, the top 10 quantitative features were listed in Table 2. On the training cohort, the highest AUC of 0.849 ± 0.027 was reached, with a sensitivity of 0.790 ± 0.038 and a specificity of 0.770 ± 0.043, respectively. Using the optimal feature set (all the 10 features), the trained model achieved an AUC of 0.849 ± 0.008 on the validation cohort, with a sensitivity of 0.724 ± 0.035, a specificity of 0.761 ± 0.017, and an accuracy of 0.743 ± 0.022, respectively.

### 3.5. IDH1 Mutation Prediction with a Fusion Model with Optimal VASARI and Radiomics Features

**Figure 5:**
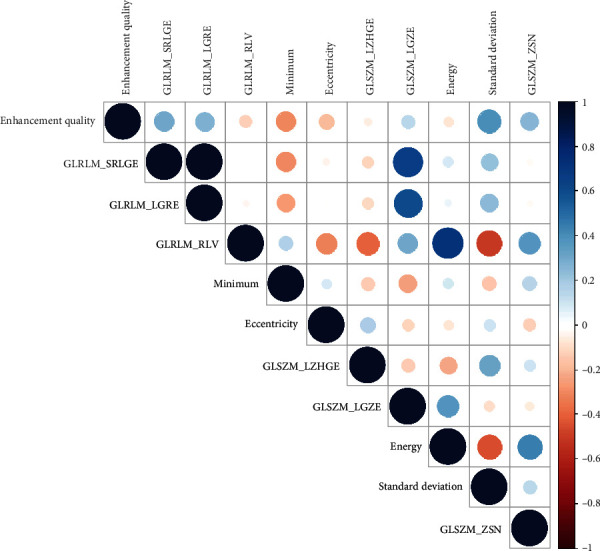
Correlation between VASARI and radiomics features in the fusion model.

**Figure 6:**
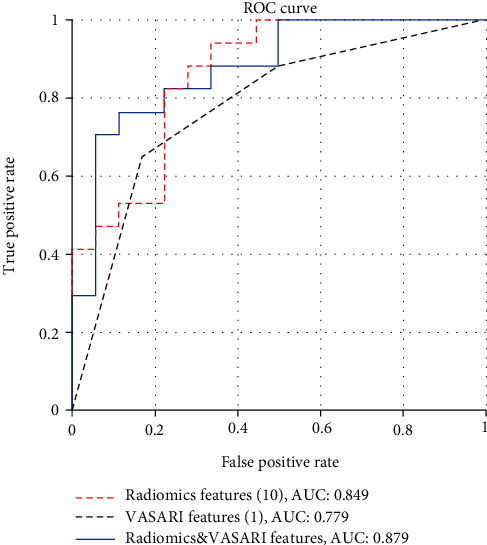
ROC curves of the constructed VASARI model, radiomics model, and the fusion model. The fusion prediction model improved the AUC to 0.879 on the validation dataset.

The fusion model was constructed with the optimal VASARI model (enhancement quality) and radiomics model (the top 10 radiomics features). Results of the Pearson correlation analysis showed that these two types of features remained very low correlation (Figure 5), demonstrating their complementary roles in the fusion model. The fusion model improved the AUC to 0.879, with a sensitivity of 0.765, a specificity of 0.778, and an accuracy of 0.771, respectively. ROC curves of the optimal VASARI model, radiomics model, and the fusion model with VASARI and radiomics features are illustrated in Figure 6. The inclusion of clinical variables including age and gender to the model did not benefit the prediction performance (AUC = 0.859).

## 4. Discussion

In this study, the machine learning algorithm was used to explore the predictive value of VASARI features based on preoperative conventional MRI images and the radiomics features based on ADC maps in IDH1 genotyping of LGG patients. The results obtained by random forest classifiers showed that the AUCs were 0.779 and 0.849 on the optimal VASARI and radiomics feature sets, respectively, and the fusion model with both feature sets achieved an improved AUC of 0.879 on the validation.

MRI is one of the essential methods for preoperative glioma diagnosis. Different imaging sequences can reveal different characteristics of tumor texture, blood supply, border, edema, hemorrhage, etc., and these characteristics are extremely important for the final diagnosis. The VASARI lexicon extracts features from routine MRI and provides standardized visual grading of MRI findings. In our study, enhancement quality was the most significant one for IDH1 mutation prediction among all VASARI features. IDH1-wildtype LGGs tended to represent a higher degree of contrast enhancement on the postcontrast T1W images compared with IDH1-mutant LGGs, which is consistent with previous studies [15–17]. Kickingereder et al. [18] found that IDH1-wildtype gliomas showed increased HIF1A activation, thus leading to a transcriptome signature induced by upregulating vasculo- and angiogenesis-related signaling pathways. Increase in proangiogenic molecules could result in more contrast agent uptake and more marked contrast enhancement on postcontrast T1W images. Besides enhancement quality, other VASARI features of strong predictive power for IDH1 mutation status included deep white matter invasion, tumor location, proportion of necrosis, and T1/FLAIR ratio. These findings are in line with those from previous studies [6, 15, 19, 20]. Among these features, tumor location in the frontal lobe in IDH1-mutant gliomas has been reported by many investigators in existing literature [21]. The frontal lobe predominance of IDH1-mutant gliomas may be because this type of tumors probably originates from glial progenitors in the forebrain subventricular zone [22]. VASARI-based random forest classifier showed an AUC of 0.779 on validation in predicting IDH1 mutation in LGGs, similar to the result reported by Park et al., who constructed a multivariable model with an AUC of 0.778 [20].

Radiomics is a method to extract quantitative features that are difficult to detect by human eyes from medical images and to use data mining and machine learning algorithms for diagnostic decision-making. In this study, radiomics analysis of ADC maps was conducted by extracting 57 quantitative features and subsequently building a prediction model with 10 optimal features. Given that the choice of classifier depends on the specific task as well as disease type, thus, comparative experiments were conducted, and ultimately random forest was chosen with the best performance for IDH1 prediction. The prediction performance on the independent validation set using different classifiers is shown in Supplementary Figure S1. Our optimal radiomics model achieved an AUC of 0.849 for IDH1 prediction in LGGs. ADC was used for radiomics analysis in our study, since ADC has been established as the most commonly used quantitative MRI metric, thus enabling first-order statistical features comparable between individuals. Previous studies have shown the benefit of ADC first-order statistical features in identifying IDH1 genotypes [23–25]. Our study further demonstrated the added value of ADC high-order radiomics features to first-order features for this purpose. Additionally, radiomics on other MRI modalities has also been investigated in terms of its relationship with IDH1 mutation status. Zhou et al. [6] found that random forest analysis of T2W-based texture features could predict IDH1 mutation status in LGGs with an AUC of 0.86, a sensitivity of 0.75, and a specificity of 0.89. By performing radiomics analysis on FLAIR images, Yu et al. [26] reported AUCs of 0.86 and 0.79 on the training and validation cohorts, respectively, in IDH1 prediction of LGGs. Interestingly, these results are consistent with ours on ADC radiomics analysis.

The major strength in our study design was the model building using both qualitative semantic and quantitative radiomics features, which were usually separately investigated in some previous studies [20, 27]. Results showed that the fusion model that integrated outputs of the optimal VASARI model and ADC-based radiomics model improved the AUC to 0.879 in IDH1 genotype prediction of LGGs, indicating that the fusion model was superior to the model using a single type of features. These findings suggest that radiomics analysis may add value to routine qualitative image analysis for IDH1 classification. Similarly, a recent study [28] also showed that the VASARI feature combined with ADC texture analysis could improve the accuracy of IDH1 mutation detection in anaplastic gliomas. In this study, although the mean age of patients with IDH1-wildtype LGG was higher than that of patients with IDH1-mutant LGG (47.3 years vs. 43.2 years), there was no statistical difference between the two groups (*P* = 0.201, independent sample *t*-test). Therefore, the inclusion of age factor in the final model failed to improve the accuracy of LGG IDH1 genotype identification.

Recently, with its rapid advancement in various fields within the past few years, deep learning has gained particular attention in the radiology domain. For example, Chang et al. [29] has used a deep learning method implemented with convolutional neural networks to classify genetic mutations in gliomas and a high accuracy of 0.94 in IDH mutation prediction was reached. Deep learning is advantageous in that it does not need human-derived feature extraction or prior feature selection [29]. However, big data are essential for a robust training process. A head-to-head comparison between conventional machine learning and deep learning methods is warranted in the future.

Apart from the intrinsic limitations of any retrospective study, several other limitations are discussed as follows. First, the cases were collected from a single center, and the patient population was relatively small. Further validation on diverse large data sets acquired from multiple vendors and across different centers is needed. Second, the numbers of included IDH1-mutant and IDH1-wildtype patients were similar (50 : 52), which did not reflect the actual prevalence of IDH mutation in LGG (around 80%) [3]. However, a balanced sampling could contribute to the model training process. Third, radiomics analysis was not performed on other routine MRI modalities. Routine MRI data were used to extract semantic features, as is performed in clinical routine. However, the results on ADC maps were consistent with those on T2W images or FLAIR images reported before [6, 26]. Advanced MRI techniques such as perfusion-weighted imaging and magnetization transfer imaging were also not adopted for radiomics analysis. The inclusion of advanced MRI modalities could provide more comprehensive functional and metabolic information and should be considered in further studies. Fourth, interobserver agreement of image segmentation was evaluated in our study. However, interobserver agreement of features was not analyzed, although it has proven to be satisfactory for both VASARI and radiomics features in previous studies [6, 20]. Fifth, considering the small sample size of our study, we did not perform weight optimization in order to avoid overfitting of the training data. Although this weighting method may lose a little performance improvement (not always), we believe that the fusion results would be more robust, especially for new data, without performance bias. It can be seen from the results that our weighted average fusion strategy played a positive role in guiding the overall forecast performance. Last, according to the 2016 WHO classification of Tumors of the Central Nervous System, 1p/19q codeletion is also an important prognostic marker in molecular diagnosis of LGGs [4]. In the study, 1p/19q codeletion status was not evaluated because this information was not available on most subjects due to the retrospective nature.

## 5. Conclusion

In conclusion, preoperative MRI VASARI features and ADC radiomics features can effectively predict IDH1 mutation status in LGG, and the fusion model integrating both predictive features shows even better prediction performance. The proposed image-based machine learning approach may provide an alternative to the conventional workflow for the noninvasive identification of IDH1 genotypes. However, these findings should be validated in large multicenter data sets in future studies.

## Figures and Tables

**Table 1:** Patient characteristics.

Characteristic	Whole cohort (*n* = 102)	Training cohort (*n* = 67)	Validation cohort (*n* = 35)	*P* value^∗^
Age (years)^†^	45.3 ± 16.3	45.7 ± 17.1	44.6 ± 14.9	0.75
Gender				
Male	60 (58.8%)	38 (56.7%)	22 (62.9%)	0.55
Female	42 (41.2%)	29 (43.3%)	13 (37.1%)	
WHO grade				
II	61 (59.8%)	44 (65.7%)	17 (48.6%)	0.10
III	41 (40.2%)	23 (34.3%)	18 (51.4%)	
IDH1 status				
Mutant	50 (49.0%)	33 (49.3%)	17 (48.6%)	0.95
Wildtype	52 (51.0%)	34 (50.7%)	18 (51.4%)	

Unless otherwise specified, data are counts (percentages). WHO: World Health Organization; IDH 1: isocitrate dehydrogenase 1. ^†^Data are means ± standard deviations. ^∗^*P* value was obtained by comparing each variable between training and validation cohorts.

**Table 2:** List of selected VASARI and radiomics features.

Feature selection			
Top 5 VASARI features	AUC value	Top 10 radiomics features	AUC value
Enhancement quality	0.752	GLRLM short run low gray-level emphasis	0.756
Deep white matter invasion	0.738	GLRLM low gray-level run emphasis	0.682
Tumor location	0.684	GLRLM run-length variance	0.678
Proportion of necrosis	0.682	Histogram minimum	0.677
T1/FLAIR ratio	0.632	Eccentricity	0.662
		GLSZM large zone high gray-level emphasis	0.641
		GLSZM low gray-level zone emphasis	0.628
		Histogram energy	0.616
		Histogram standard deviation	0.612
		GLSZM zone-size nonuniformity	0.607

VASARI: Visually Accessible Rembrandt Images; AUC: area under the receiver operating characteristic curve; GLRLM: gray-level run-length matrix; GLSZM: gray-level size zone matrix.

## Data Availability

The datasets used and/or analyzed during the current study are available from the corresponding author on reasonable request.

## Supplementary Materials

Supplementary Materials Table S1: summary of VASARI features. Table S2: summary of radiomics features. Figure S1: ROC curves show the prediction performance for IDH1 mutation on the independent validation set using different classifiers. Random forest classifier showed the best prediction performance with an area under the ROC curve of 0.849. RF: random forest; SVM: support vector machine; LDA: linear discriminant analysis; KNN: k-nearest neighbor.
