# Supplementary material for: Qualitative and Quantitative MRI Analysis in IDH1 Genotype Prediction of Lower-Grade Gliomas: A Machine Learning Approach
Source: Biomed Res Int. 2021 Jan 22;2021:1235314. doi: 10.1155/2021/1235314 (PMC7847347; doi:10.1155/2021/1235314)
Supplement: Supplementary Materials — Table S1: summary of VASARI features. Table S2: summary of radiomics features. Figure S1: ROC curves show the prediction performance for IDH1 mutation on the independent validation set using different classifiers. Random forest classifier showed the best prediction performance with an area under the ROC curve of 0.849. RF: random forest; SVM: support vector machine; LDA: linear discriminant analysis; KNN: k-nearest neighbor. [file 1235314.f1.docx]

**Table S1.** Summary of VASARI Features.

| Number | Name | Options |
| --- | --- | --- |
| F1 | Tumor location | Frontal; Temporal; Insular; Parietal; Occipital; Other |
| F2 | Side of tumor epicenter | Right; Center/Bilateral; Left |
| F3 | Enhancement quality | None; Mild/Minimal; Marked/Avid |
| F4 | Proportion enhancing | 0; 1~33%; >33% |
| F5 | Proportion necrosis | 0; 1~33%; >33% |
| F6 | Cyst | No; Yes |
| F7 | Multifocal or multicentric | No; Yes |
| F8 | T1/FLAIR ratio | T1~FLAIR; T1<FLAIR/ T1<<FLAIR |
| F9 | Thickness of enhancing margin | None; Thin; Thick/solid; N/A |
| F10 | Definition of enhancing margin | Well-defined; Poorly-defined; N/A |
| F11 | Definition of non-enhancing margin | Smooth; Irregular |
| F12 | Proportion of edema | 0; 1~33%; >33% |
| F13 | Edema crosses midline | No; Yes |
| F14 | Hemorrhage | No; Yes |
| F15 | Pial invasion | No; Yes |
| F16 | Ependymal invasion | No; Yes |
| F17 | Cortical involvement | No; Yes |
| F18 | Deep white matter invasion | No; Yes |
| F19 | Non-enhancing tumor crosses midline | No; Yes |
| F20 | Enhancing tumor crosses midline | No; Yes |
| F21 | Satellites | No; Yes |
| F22 | Calvarial remodeling | No; Yes |
| F23 | Lesion size (cm) | ≤2; >2&≤4; >4&≤6; >6&≤8; >8 |

VASARI = Visually Accessible Rembrandt Images

N/A = Not applicable

**Table S2.** Summary of Radiomics Features.

| Type | Name |
| --- | --- |
| Shape features (*n* = 3) | Volume, Eccentricity, Solidity |
| First-order histogram features (*n* = 13) | Mean, Standard deviation, Skewness, Kurtosis, Energy, Entropy, Min, Max, 10%, 25%, 50%, 75%, 90% percentiles |
| Gray-level co-occurrence matrix (GLCM) features (*n* = 9) | Energy, Contrast, Entropy, Homogeneity, Correlation, SumAverage, Variance, Dissimilarity, AutoCorrelation |
| Gray-level run-length matrix (GLRLM) features (*n* = 13) | Short Run Emphasis (SRE), Long Run Emphasis (LRE), Gray-Level Non-uniformity (GLN), Run-Length Non-uniformity (RLN), Run Percentage (RP), Low Gray-Level Run Emphasis (LGRE), High Gray-Level Run Emphasis (HGRE), Short Run Low Gray-Level Emphasis (SRLGE), Short Run High Gray-Level Emphasis (SRHGE), Long Run Low Gray-Level Emphasis (LRLGE), Long Run High Gray-Level Emphasis (LRHGE), Gray-Level Variance (GLV), Run-Length Variance (RLV) |
| Gray-level size zone matrix (GLSZM) features (*n* = 13) | Small Zone Emphasis (SZE), Large Zone Emphasis (LZE), Gray-Level Non-uniformity (GLN), Zone-Size Non-uniformity (ZSN), Zone Percentage (ZP), Low Gray-Level Zone Emphasis (LGZE), High Gray-Level Zone Emphasis (HGZE), Small Zone Low Gray-Level Emphasis (SZLGE), Small Zone High Gray-Level Emphasis (SZHGE), Large Zone Low Gray-Level Emphasis (LZLGE), Large Zone High Gray-Level Emphasis (LZHGE), Gray-Level Variance (GLV), Zone-Size Variance (ZSV) |
| Neighborhood gray-tone difference matrix (NGTDM) features (*n* = 5) | Coarseness, Contrast, Busyness, Complexity, Strength |


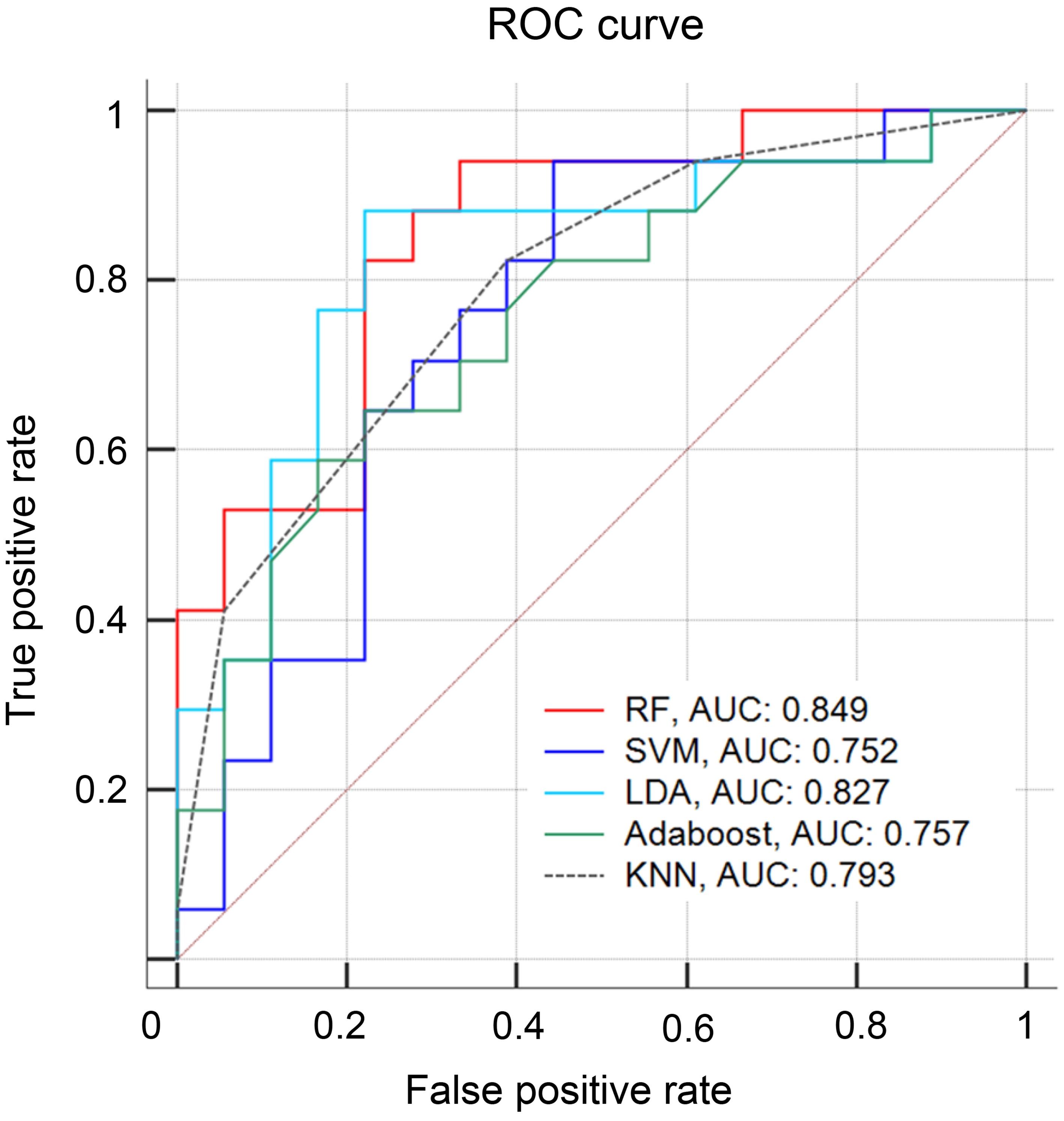


**Figure S1.** ROC curves show the prediction performance for IDH1 mutation on the independent validation set using different classifiers. Random forest classifier showed the best prediction performance with an area under the ROC curve of 0.849. RF = random forest, SVM = support vector machine, LDA = linear discriminant analysis, KNN = k-nearest neighbor.
